# Some Deafness-Causing Mutations Can Be Silenced with the Appropriate Gene Partner

**DOI:** 10.1100/tsw.2001.40

**Published:** 2001-05-01

**Authors:** Edward Wilcox, Saima Riazuddin, Sheikh Riazuddin

**Affiliations:** Laboratory of Molecular Genetics, NIDCD, NIH, Rockville, MD 20850-3227, USA

**Keywords:** deafness, linkage analysis, modifier-genes

## Abstract

Inheritance of a deafness-causing genotype does not necessarily mean that a person will have profound hearing loss. The presence of a modifying gene can change the effect of the deafness genotype. As an example, mutations found commonly among the deaf are at times found among their normal hearing relatives [1]. The implication is that there are genes or gene products whose interactions allow the normal physiological function of the inner ear despite a mutation that would normally disrupt the process. Deafness is not the first disorder in which modifiers can change the expected outcome, nor will it be the last, but it is very unusual for the outcome to be so dramatically changed.

Of the 3.9 million children born every year in the U.S. [2], 1 in 1000 has a profound hearing deficit [3]. The percentage of children born with hearing loss is probably higher in countries such as Pakistan, due in part to the custom of marriage among first cousins [4]. The Library of Congress describes this custom as follows: “Marriage with one’s father’s brother’s child is preferred, in part because property exchanged at marriage then stays within the patrilineage. If a marriage is successful, it will be followed by others between the two families” [5].

Studying large interrelated families with inherited deafness can simplify the study of hearing loss in several ways: 1) in most cases, the hearing loss inherited in a family can be attributed to a single gene; 2) large families provide high statistical power because of many children over many generations; 3) if a gene product interaction exists that modifies or changes the outcome of profound hearing loss, it can be documented in the many branches of an extended family just as has been done for several mouse models of hereditary deafness [1].

In Pakistan and other regions with similar marriage practices, extended families have a limited gene pool and can be considered population isolates. Such families provide an important tool in mapping and identifying recessive genes. Eight deaf kindred in one such family with 141 members over eight generations were found linked to markers on chromosome 4 (now called *DFNB26*) [6]. Surprisingly, seven additional normal hearing members of the family also had the “disease genotype”. Upon close examination, none of these seven kindred had any evidence of hearing loss.

To support the hypothesis of a gene modifier, many additional markers were mapped throughout the genome. The whole genome, except markers around the deafness-causing *DFNB26* mutation on chromosome 4, could be excluded as a cause of this inherited deafness with high statistical power. At the same time, it was noted that markers on chromosome 1 inherited from one female progenitor provided full protection against the recessive hearing loss gene on chromosome 4. The deafness modifier (*DFNM1*) protected her granddaughter and six of her great-grandchildren from hearing loss.

With the complete sequence of the human genome now in hand, it shouldn't be long before both genes (*DFNM1* and *DFNB26*) on chromosome 1 and 4, respectively, are identified and the nature of their interaction discovered. Understanding this interaction could lead to new insights regarding the physiology of hearing and the nature of protein interactions that lead to the protection of hearing in the presence of deleterious genes.

| | | |
|---|---|---|
| Received: | March | 6, 2001 |
| Accepted: | March | 26, 2001 |
| Published: | May | 1, 2001 |
